# Preparation and In Vitro Characterization of Quercetin and Chlorhexidine-loaded PLGA Nanoparticles for Oral Biofilm-Associated Pathologies

**DOI:** 10.1007/s00784-026-06954-0

**Published:** 2026-06-26

**Authors:** İskender İnce, Yeliz Yıldırım, Gülnur Emingil, Barış Gümüştaş, Nil Yakar, Güven Özdemir, Alpdoğan Kantarcı

**Affiliations:** 1 Center for Drug Research and Pharmacokinetic Applications (ARGEFAR), Ege University, Izmir, Turkey; 2 Institute of Nuclear Sciences, Department of Nuclear Applications, Ege University, Izmir, Turkey; 3 Faculty of Science, Department of Chemistry, Ege University, Izmir, Turkey; 4 Faculty of Dentistry, Department of Periodontology, Istinye University, Istanbul, Turkey; 5 Faculty of Science, Department of Biology, Basic and Industrial Microbiology Section, Ege University, Izmir, Turkey; 6 Department of Developmental and Surgical Sciences, University of Minnesota, Minneapolis, MN USA; 7 Department of Oral Medicine, Infection and Immunity, Harvard School of Dental Medicine, Boston, MA USA

**Keywords:** Quercetin, Chlorhexidine, Periodontal Diseases, Poly(lactic-co-glycolic acid)

## Abstract

**Objectives:**

This study aimed to develop and evaluate a dual-drug-loaded PLGA nanoparticle system incorporating quercetin (QUE) and chlorhexidine (CHX) for localized, sustained delivery, with potential application in biofilm-associated pathologies.

**Materials and methods:**

Single- and dual-drug systems containing CHX and QUE at different concentrations (1.5%, 5%, and 15%) were successfully loaded into poly(lactic-co-glycolic acid) nanoparticles (PLGA NPs) with high encapsulation efficiency. Physicochemical characterization was performed using dynamic light scattering (DLS), zeta potential analysis, SEM-EDX, FTIR, and thermal analysis (DSC and TGA). Release kinetics of QUE- and CHX-loaded nanoparticles were evaluated in an artificial saliva environment, and the amounts of CHX and QUE released were quantified by high-performance liquid chromatography (HPLC). Antimicrobial activity was assessed against *Staphylococcus aureus* and *Escherichia coli* using the disc diffusion method.

**Results:**

The prepared nanoparticles displayed spherical morphology with sizes ranging from 54.08 to 356.1 nm and zeta potentials from − 2.11 to -12.46 mV, indicating colloidal stability. FTIR and thermal analysis confirmed molecular dispersion of drugs and polymer-drug interactions. QUE showed complete release within 168 h in the single-drug system, whereas co-loading with CHX extended QUE retention, with 20% remaining after 240 h. CHX release reached ∼80% in both formulations. CHX/QUE nanoparticles demonstrated superior antibacterial activity compared to QUE-only systems, effectively inhibiting both Gram-positive and Gram-negative bacteria.

**Conclusion:**

These compounds and formulations are designed for clinical applications due to their slow, controlled release of the dual-active PLGA NP system.

## Introduction

Biofilm-associated pathologies pose a major challenge in clinical practice due to their increased resistance to antimicrobial agents and host defense mechanisms, underscoring the need for advanced drug delivery systems that enhance antimicrobial efficacy. Periodontal diseases are among the prevalent biofilm-associated inflammatory pathologies [1–4]. The failure to limit inflammatory and immune events in the periodontium results in uncontrolled immune responses, leading to chronic inflammation, tissue destruction, overproduction of inflammatory cytokines and host-derived enzymes, and loss of connective tissue and alveolar bone [5, 6]. Based on the recent classification of periodontal diseases [7], the European Federation of Periodontology (EFP) has developed Clinical Practice Guidelines (CPGs) to provide evidence-based therapeutic strategies tailored to each disease stage, emphasizing a need for local antimicrobial agents in advanced cases (Stage III and IV). Adjunctive strategies involving dual-drug therapies (e.g., combinations of antimicrobials and anti-inflammatory agents) have the potential to enhance clinical attachment levels, reduce probing depth, and lower inflammatory biomarkers [8, 9].

Dual-drug-loaded nanoparticle systems with antimicrobial and antioxidant capabilities offer synergistic advantages by targeting both microbial burden and the inflammatory process [10–13]. Polyphenol compounds (e.g., polyphenol extracts from blueberry, water lily, seaweed, and peppermint seed) have been shown to suppress planktonic periodontal pathogens [14, 15]. Quercetin (QUE) is a bioactive flavonoid widely found in various fruits and vegetables, particularly in apples, onions, and berries, with potent antioxidant, anti-inflammatory, and antimicrobial effects [14, 16–22]. QUE was shown to suppress the production of IL-1β, TNF-α, IL-17, RANKL, and ICAM-1 in the gingival tissue, suggesting that QUE prevents periodontitis by modulating cytokines and ICAM-1 [20]. In addition, QUE was shown to be effective in the structure and metabolic activity of mature, pathogenic biofilms [14]. Although specific pathogenic bacteria in mature biofilms were removed, *S. mitis* was less susceptible [14]. QUE showed potent antimicrobial activity against A. actinomycetemcomitans, P. gingivalis, and other oral pathogens [18, 19]. Meanwhile, chlorhexidine (CHX) remains the gold-standard broad-spectrum antimicrobial in dentistry; however, its long-term use is associated with several limitations [23, 24]. Water-soluble CHX digluconate salt is used daily as a mouthwash for gingivitis, to prevent ventilator-associated pneumonia, as an irrigant in endodontic treatment, and to treat oral mucositis [25–29]. CHX is effective against gram-positive and gram-negative bacteria, fungi, and viruses [30]. Its effectiveness in reducing plaque and gingivitis is related to its antimicrobial properties and high substantivity, which are enhanced by its cationic nature [29]. Its antibacterial actions reduce pellicle formation, prevent bacterial adhesion to the tooth surface, and alter bacterial cell walls by increasing cell membrane permeability and coagulating intracellular cytoplasmic macromolecules [31], ultimately altering the microflora in the periodontal pocket and inhibiting microbial proteases of periodontal pathogens [32]. However, studies using confocal laser scanning microscopy have demonstrated that the antimicrobial effect of CHX on mature dental biofilms is largely superficial, with limited penetration into deeper biofilm layers [33]. Nanoparticle-based delivery of CHX has been shown to improve its penetration and antimicrobial efficacy against mono- and multi-species oral biofilms compared to conventional formulations [34]. The most studied release device is the gelatin-based CHX-containing chip (Periochip), which is placed in a deep pocket and releases CHX for 7 days [35]. Although the preparation size is suitable for severe periodontitis, its application to the pocket dimensions in mild and moderate cases is limited [36, 37]. Accordingly, various biodegradable and locally applicable drug delivery systems have been developed to achieve sustained and site-specific release of chlorhexidine within the periodontal pocket [38]. Thus, different systems need to be tested for the controlled release of CHX into the periodontal pocket [4]. In this study, we developed and evaluated a dual-drug–loaded PLGA nanoparticle system incorporating quercetin (QUE) and chlorhexidine (CHX) to achieve localized and sustained antimicrobial and anti-inflammatory delivery for oral biofilm-associated pathologies.

## Materials and methods

### Preparation of chlorhexidine (CHX)/quercetin (QUE) loaded poly(lactic-co-glycolic acid) (PLGA) nanoparticles

The nanoprecipitation method was used to synthesize PLGA nanoparticles (PLGA NP). 80 mg of PLGA was dissolved in 2 mL of acetone. A clear solution of 4 ml of 5% polyvinyl alcohol (PVA) crosslinker was prepared in distilled water at 90^°^C. PLGA solution was added to the PVA solution under magnetic stirring for 4 h. The nanoparticles were centrifuged at 14,000 rpm for 30 min, and the upper phase was removed. The remaining precipitate was washed and vortexed 3 times by adding distilled water. CHX (1.5%, 5%, 15%), QUE (1.5%, 5%, 15%) and CHX + QUE (1.5%/1.5%, 5%/5% and 15%/15%) were added. It was frozen at -20^°^C for 15 min and then freeze-dried with a lyophilizer (Telstar Cryodos, Spain) to obtain powdered formulations. The formulations were stored at 2–6 °C, protected from light, to prevent degradation of the molecules before use. The characterization of the synthesized formulations was performed using Fourier Transform Infrared (FTIR) (PerkinElmer Spectrum 100), Dynamic Light Scattering Spectrometry (DLS), Malvern Zeta Sizer Ultra, Thermogravimetry Analysis (TG) (Perkin-Elmer Diamond), Differential Scanning Calorimetry (DSC) (Shimadzu DSC 60), and Scanning Electron Microscopy (SEM) (Thermo Scientific Apreo S SEM-EDX).

### Release kinetics of CHX and QUE in loaded PLGA Nanoparticles

The calibration curve was created by plotting peak area values against the concentrations of 6 standard solutions ranging from 0.025 to 2.50 µg/mL. To determine the efficiency of QUE encapsulated into PLGA NP, the synthesized QUE-loaded PLGA NP and CHX/QUE-loaded PLGA NPs were mixed in 20 mL of pure water at 90^°^C for 30 minutes, brought to room temperature, and 20 mL of DMSO was added and stirred for another 30 minutes. After dilution with an appropriate amount of mobile phase, it was centrifuged at 4000 rpm for 5 min and filtered. The concentration of QUE was determined by injecting 20 µL of standard into the Shimadzu LC-20A HPLC-PDA (High-Performance Liquid Chromatography). Avantor ACE 5 C-18-AR (250 * 4.6) analytical column was used for separation, and a mixture of acetonitrile and 2% acetic acid containing water (pH = 3) (60% − 40% v/v) was used as the mobile phase. Since the QUE retention time was approximately 7 minutes, the total analysis time was 10 minutes. The working conditions for the method were wavelength = 370 nm, flow rate = 1.3 mL/min, column oven temperature = 35°C, and injection volume = 20 µL [39]. CHX concentration was determined by injecting 20 µL of standard into the Shimadzu LC-20A HPLC-PDA. Avantor Zorbax Eclipse XDB-C18 (150 * 4.6) analytical column was used for separation, and a mixture of acetonitrile and 0.04 M phosphate buffer (pH: 3) (% 70 - % 30 v: v) was used as the mobile phase. Since the retention time for CHX was approximately 5 minutes, the total analysis time was set to 10 minutes. The working conditions for the method were wavelength = 239 nm, flow rate = 1.0 mL/min, column oven temperature = 40°C, and injection volume = 20 µL [40]. To ensure the reliability of the analyses, a ‘Partial Analytical Method Validation’ study was conducted, and the compliance of the validation parameters with the criteria established before the study was examined. The calibration curve was created by plotting peak area values against the concentrations of 6 standard solutions ranging from 0.2 to 10.0 µg/mL.

Then, in vitro release profiles of CHX-loaded PLGA, QUE-loaded PLGA, and CHX/QUE-loaded PLGA nanoparticles were performed in an artificial saliva medium to reproduce the oral environment. 5 mg CHX and/or QUE-loaded PLGA samples were placed in 10 mL of pH = 6.8 artificial saliva medium at 37 °C. A 0.1 mL sample of medium was taken, and CHX and QUE were determined using the HPLC methods described above. At each time, 0.1 mL of medium was replaced with fresh artificial saliva [41]. Stock solutions of 0.1 M CaCl_2_.H_2_O (A) and 0.3 M NaH_2_PO_4_.H_2_O (B) were prepared. Before each release experiment, 10 mL of stock solutions (A) and (B) were freshly taken, and 1.68 g of NaHCO3 was added to each solution, which was diluted to 1 L with distilled water and then filtered through filter paper.

### Stability of the CHX/QUE-loaded PLGA nanoparticles

The stability studies of the prepared nanoparticles were conducted in accordance with the stability guide. The systems were stored at 4 ± 2 °C (in the refrigerator), 25 ± 2 °C (60 ± 5% relative humidity), and 40 ± 2 °C (75 ± 5% relative humidity). Samples were checked at 0, 1, 2, 3, and 6 months. The amount of active ingredient in CHX-loaded PLGA, QUE-loaded PLGA, and CHX/QUE-loaded PLGA nanoparticles was determined by HPLC at the specified time intervals, and the active ingredient stability was evaluated.

### Antimicrobial activity of CHX/QUE-loaded PLGA nanoparticles

UV application did not affect the morphology or arrangement of the synthesized nanoparticles during sterilization, as reported previously for PLA-based nanostructures [42]. Therefore, UV irradiation, which is widely used as a convenient sterilization method for polymeric nanoparticle systems, was applied prior to antibacterial studies for CHX-loaded PLGA, QUE-loaded PLGA, and CHX/QUE-loaded PLGA nanoparticles [43]. Each side of CHX-loaded PLGA, QUE-loaded PLGA, and CHX/QUE-loaded PLGA nanoparticles was sterilized under a UV lamp (254 nm) in a sterile cabinet for 30 min. FTIR and TG analyses were performed to determine whether degradation occurred after sterilization.

Organisms and culture conditions were CHX-loaded PLGA, QUE-loaded PLGA, and CHX/QUE-loaded PLGA nanoparticles containing 5% active substance, as well as free CHX and free QUE. Their antimicrobial effects on Escherichia coli (ATCC 10536) and Staphylococcus aureus (ATCC 6538) were investigated. Bacteria cultured on tryptic soy broth (TSB) (Neogen, UK) and tryptic soy agar (TSA) (Neogen, UK) were incubated for 24 h at 37 °C under aerobic conditions.

CHX-loaded PLGA, QUE-loaded PLGA, and CHX/QUE-loaded PLGA nanoparticles, QUE (5 mg/mL), and CHX (0.1 mg/mL) were prepared by dissolving in 100% DMSO, and these solutions were sterilized using a 0.2 Νm polytetrafluoroethylene membrane filter (Minisart SRP, Sartorius).

A modified Kirby-Bauer method was used to determine antimicrobial susceptibility. In this process, the agar contact method was performed [44]. The liquid culture cultivated the night before was adjusted to 0.5 McFarland turbidity and spread onto 0.1 ml TSA plates. CHX-loaded PLGA, QUE-loaded PLGA, and CHX/QUE-loaded PLGA nanoparticles, QUE (5 mg/mL), and CHX (0.1 mg/mL) solutions were serially diluted 12 times at a ratio of 1/2. 100% DMSO solution was used as a control. All conditions were dropped onto freshly cultivated plates at 10 𝜇L. After a 24-hour incubation at 37 °C, bacterial susceptibility was considered positive for substances and concentrations that produced an inhibition zone of 3 mm or wider. The lowest concentration that created the inhibition zone was recorded as the minimum effective concentration (MEC).

The microdilution method was used to determine the minimum inhibitory concentration (MIC). The CHX-loaded PLGA, QUE-loaded PLGA, CHX/QUE-loaded PLGA nanoparticles, QUE (1.6 mg/mL), and CHX (0.8 mg/mL) solutions obtained by diluting the stock solution with phosphate buffer solution were serially diluted 12 times with 50% 4% DMSO solvent. The liquid culture was diluted to approximately 10^6^ colony-forming units. 100 𝜇L of bacterial inoculum was added to microwells containing 100 𝜇L of CHX-loaded PLGA, QUE-loaded PLGA, CHX/QUE-loaded PLGA nanoparticles, QUE, CHX, and 4% DMSO as a control and incubated for 24 h at 37 °C. Minimum inhibitory concentrations were interpreted as wells with no visual turbidity.

Minimum cidal concentration (MSC) was determined by incubating aliquots above the MIC by dropping 10 𝜇L onto TSA plates. The relevant concentration was considered bactericidal when Petri plates showed no growth after incubation at 37 °C for 24 h. All experiments were performed in triplicate (*n* = 3).

## Results

### Characterization of nanoparticles

**Fig. 1:**
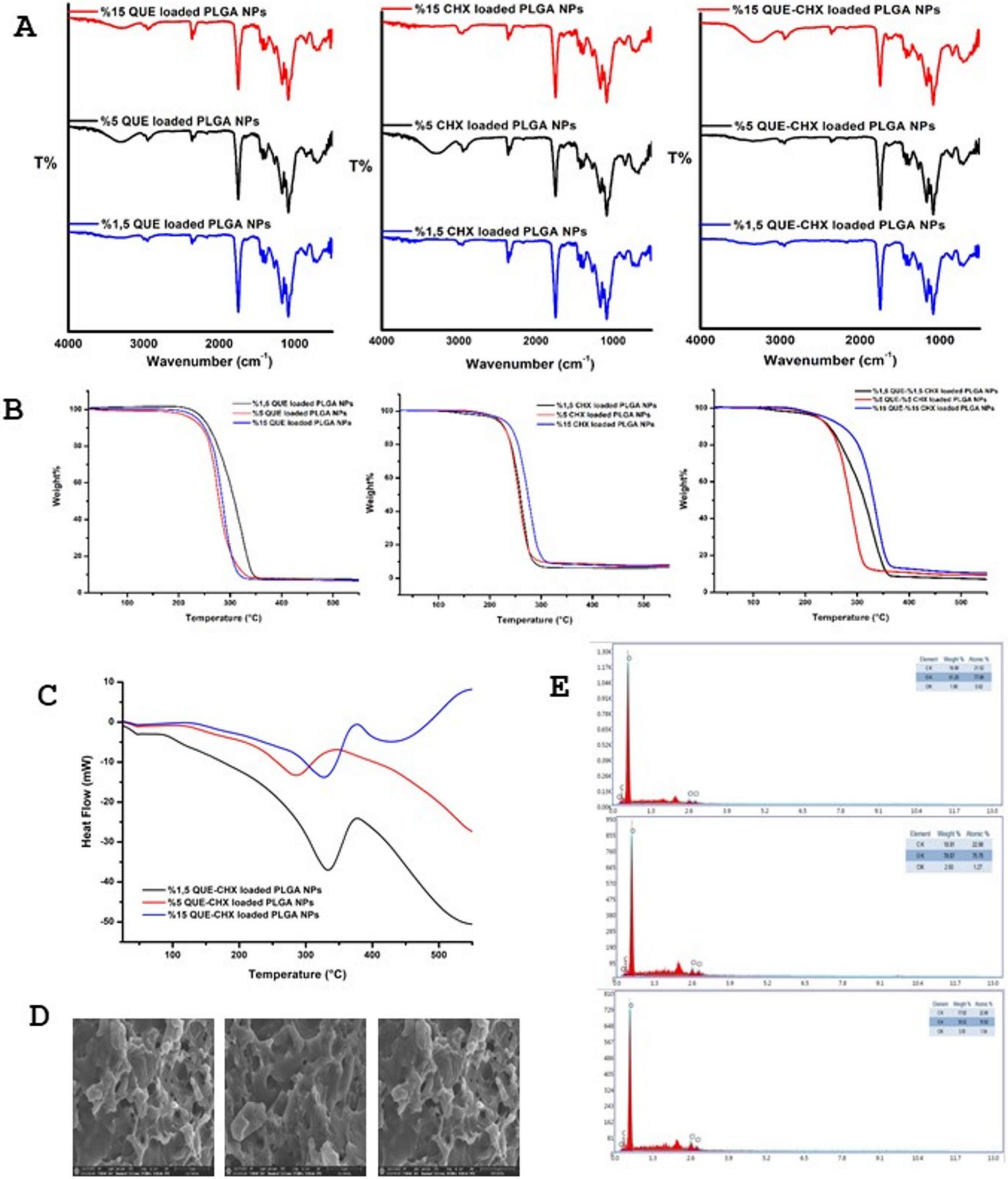
**(A)** FTIR spectra of 1.5%, 5%, 15% QUE and/or CHX loaded PLGA NP, **(B)** TG thermograms of 1.5%, 5%, 15% QUE and/or CHX loaded PLGA NP, **(C)** DSC thermograms of 1.5%, 5%, 15% QUE and/or CHX loaded PLGA NP, **(D)** SEM imaging of 1.5%, 5%, 15% QUE and/or CHX loaded PLGA NP, **(E)** SEM-EDX results of 1.5%, 5%, 15% QUE and/or CHX loaded PLGA NP

The chemical characterization of the prepared nanoparticles was investigated by FTIR (Fig. 1A), size measurements, and zeta potential measurements by DLS (Table 1), thermal behaviors by TG (Fig. 1B), DSC (Fig. 1C), and surface morphology by SEM/SEM-EDX (Figs. 1D **and E**). FTIR analyses revealed chemical interactions among QUE, CHX, and PLGA NPs. The FTIR spectra were recorded over 500–4000 cm-1, with 10 repetitions at 4 cm-1 resolution, using a KBr pellet. Figure 1A shows the FTIR spectra of 1.5%, 5%, 15% QUE-loaded PLGA NP, 1.5%, 5%, 15% CHX-loaded PLGA NP, and 1.5/1.5%, 5/5%, 15/15% CHX/QUE-loaded PLGA NP, respectively. To elucidate the chemical structures of nanoparticle systems, the FTIR spectra in Fig. 1A (first spectrum) show the characteristic bands of PLGA NP, which are the absorbance bands of -CH, -CH_2_, -CH_3_ stretching at 2850–3000 cm^− 1^ wave number, –C = O stretching at 1750–1800 cm^− 1^, stretching vibrations of C-O bonds at 1050–1250 cm^− 1^ and -OH stretching vibrations at 3200–3600 cm^− 1^ [45, 46]. In the FTIR spectra of QUE-loaded PLGA nanoparticles, absorption bands corresponding to O–H stretching vibrations in the range of 3500–3400 cm⁻¹, a C = O stretching band at approximately 1662 cm⁻¹, and C–C stretching bands around 1618 cm⁻¹ were observed. With increasing QUE concentration, the O–H stretching band exhibited a reduction in intensity and a slight shift in wavenumber [47, 48].

**Table 1:** Blank and 1.5%, 5%, and 15% drug-loaded PLGA NP size and zeta potential measurement results

Sample Code	Particle size (nm)	Zeta potential (mV)
PLGA NP	85,00	–21,17
%1,5 QUE loaded PLGA NP	66,87	–2,11
%5 QUE loaded PLGA NP	70,53	–7,65
%15 QUE loaded PLGA NP	110,80	–12,10
%1,5 CHX loaded PLGA NP	54,08	–4,39
%5 CHX loaded PLGA NP	64,85	–8,66
%15 CHX loaded PLGA NP	159,20	–9,96
1.5/1.5% CHX/QUE loaded PLGA NP	95,65	–2,16
5/5% CHX/QUE loaded PLGA NP	171,00	–6,86
15/15% CHX/QUE loaded PLGA NP	356,10	–12,46

CHX exhibited characteristic absorption bands at approximately 1090 cm⁻¹ and 3300 cm⁻¹, corresponding to C–N and N–H bending vibrations, respectively [49]. The FTIR spectrum of PLGA nanoparticles displayed characteristic ester and carbonyl absorption bands at 1100–1250 cm⁻¹ and 1750–1760 cm⁻¹ [50]. In the FTIR spectra of PLGA NPs loaded with both QUE and CHX (Fig. 1A, third spectrum), characteristic peaks of CHX at 1093 cm^− 1^ (C-N bond) and QUE at 1662 cm^− 1^ (C = O bond) were observed. The size and zeta potential measurement results of blank and 1.5%, 5%, and 15% drug-loaded PLGA NP samples obtained with the DLS device are given in Table 1.

Particle size and surface charge characteristics were determined using Dynamic Light Scattering (DLS). The average hydrodynamic diameters ranged from 54.08 to 356.10 nm, with polydispersity indices (PDIs) below 0.3, indicating homogeneous nanoparticle populations suitable for systemic delivery. Zeta potential values ranged between − 2.11 and − 12.46 mV, suggesting moderate electrostatic stability. Notably, dual-loaded CHX/QUE nanoparticles exhibited intermediate zeta potentials, indicating successful co-encapsulation and potential drug-drug interactions that affect surface characteristics.

Figure 1B shows the TG termograms of 1.5%, 5%, 15% QUE loaded PLGA NP, 1.5%, 5%, 15% CHX loaded PLGA NP, and 1.5/1.5%, 5/5%, 15/15% CHX/QUE-loaded PLGA NP, respectively. The relationship between temperature increase and mass loss in the nanoparticle can be determined by thermogravimetric analysis (TG). The TG thermograms of QUE-loaded PLGA NPs (Fig. 1B, first spectrum) exhibited comparable profiles across different QUE concentrations. All samples showed a single-step decomposition starting at approximately 250 °C and ending at 350 °C. The residual mass at 500 °C was approximately 7% for all QUE-loaded PLGA NP formulations. The TG thermograms of CHX-loaded PLGA NPs (Fig. 1B, second spectrum) showed a comparable thermal profile, with single-step degradation between 200 °C and 350 °C. According to the TG data of CHX, approximately 17% residual mass was observed at 800 °C. In this case, the residual mass values observed in the TG thermograms were 6.5% for 1.5% CHX-loaded PLGA NPs, 7% for 5% CHX-loaded PLGA NPs, and 8% for 15% CHX-loaded PLGA NPs. The TG thermograms of CHX/QUE-loaded PLGA NPs (Fig. 1B, third spectrum) exhibited comparable thermal behavior, characterized by a single-step degradation starting at approximately 200 °C and ending at 400 °C. The residual mass at 400 °C increased from 7% to 11% with increasing drug loading.

DSC analysis, which is a thermoanalytical technique used to observe crystallization events, was performed using sample amounts of 5–10 mg at a flow rate of 20 mL/min and a heating rate of 10 °C/min in an N_2_ atmosphere at a temperature range of 25–550 °C. Figure 1C shows the DSC thermograms of PLGA NPs loaded with 1.5%/1.5%, 5/5%, and 15/15% CHX/QUE. When the DSC thermograms of the prepared CHX/QUE-loaded PLGA nanoparticle systems were examined, a broad endothermic peak starting at approximately 150 °C and ending at around 380 °C was observed for the 1.5/1.5%, 5/5%, and 15/15% CHX/QUE-loaded PLGA nanoparticle formulations (Fig. 1C).

The SEM characterized the shape and surface morphology of the prepared dual drug-loaded PLGA NPs. SEM and EDX images of 1.5/1.5% CHX/QUE-loaded PLGA NP, 5/5% CHX/QUE-loaded PLGA NP, and 15/15% CHX/QUE-loaded PLGA NP samples are shown in Fig. 1D **and E**, respectively. When the SEM surface analyses of PLGA NPs loaded with different amounts of CHX/QUE at 1.5/1.5%, 5/5%, and 15/15% were examined, the amount of loaded QUE and CHX did not affect the surface morphology. QUE and CHX nanoparticles loaded at various percentages exhibit reticulated and porous structures. The presence of Cl atoms in the CHX structure and the increase in CHX loading percentage resulted in a higher Cl signal in the EDX analysis, supporting the presence and distribution of CHX within the nanoparticles and consistent with the incorporation of both CHX and QUE into the PLGA matrix.

### Determination of CHX and QUE amounts in CHX/QUE-loaded PLGA nanoparticles

**Fig. 2:**
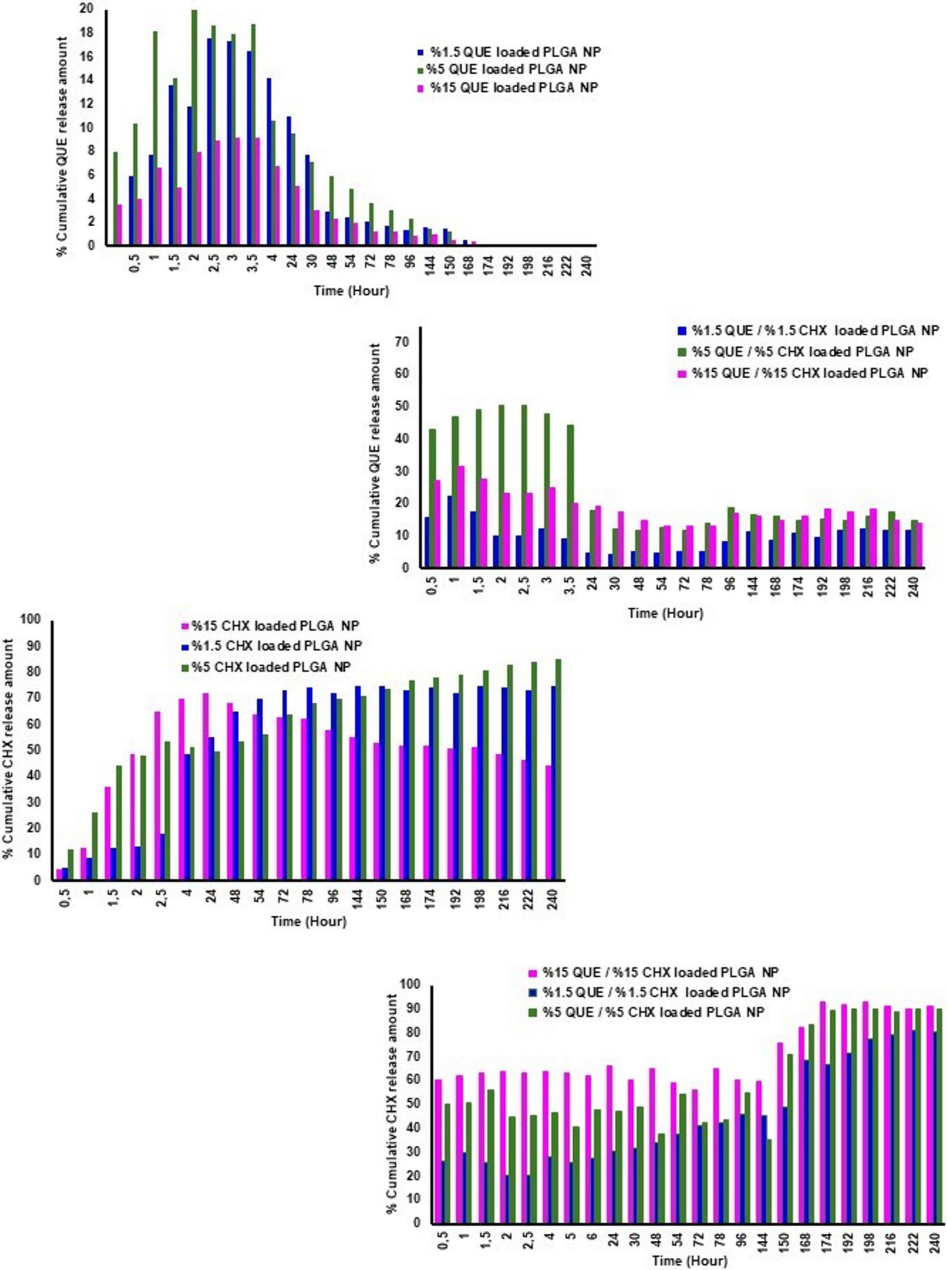
**A)** % Cumulative QUE and CHX released amount-time graph of 1.5%, 5%, 15% QUE and/or CHX loaded PLGA NP in artificial saliva

The calibration curves for the QUE and CHX were created by plotting the peak area values against the concentration values of the 6 selected standard solutions at different concentrations. The values of the regression parameters for the QUE curve were calculated with the equation y = ax + b; the values were a = 31,589, b = 12.647, and R^2^ = 0.9999. The value of the regression coefficient was deemed appropriate. The values of the regression parameters for the CHX curve were calculated with the equation y = ax + b; the values were a = 33,690, b = -534.38, and R^2^ = 0.9997. The value of the regression coefficient was deemed appropriate. All parameters were found to comply with the criteria established prior to the validation study. The cumulative release profiles of QUE and CHX from nanoparticle formulations are illustrated in Fig. 2. The maximum QUE release from single drug-loaded nanoparticles peaked at 20%, while dual-loaded CHX/QUE nanoparticles achieved up to 40% QUE release. For CHX, cumulative release rates of up to 80% were obtained in both systems, while QUE exhibited a sustained release of approximately 20% at 240 h in CHX/QUE-loaded PLGA nanoparticles. Its activity was lost after 168 h when QUE loaded PLGA NPs alone.

### In vitro stability of the CHX and QUE-loaded PLGA nanoparticles

Stability studies of the prepared nanoparticles were carried out at 4 ± 2 °C (refrigerator), 25 ± 2 °C, 60 ± 5% relative humidity (climate cabinet), and 40 ± 2 °C, 75 ± 5% relative humidity (climate cabinet) conditions. The amounts of QUE and CHX in the products were determined by HPLC at 0, 1, 2, 3, and 6 months. As a result of stability tests conducted at 25 ± 2 °C, 60 ± 5% relative humidity, and 40 ± 2 °C, 75 ± 5% conditions, gelation tendency and decreases in active ingredient amounts were observed in the products after one month. Studies at 4 ± 2 °C conditions revealed that both active ingredients were stable under these conditions. In addition, stability studies conducted at room temperature showed that the products were stable for 11 days.

### UV sterilization of the CHX and QUE-loaded PLGA nanoparticles

**Fig. 3:**
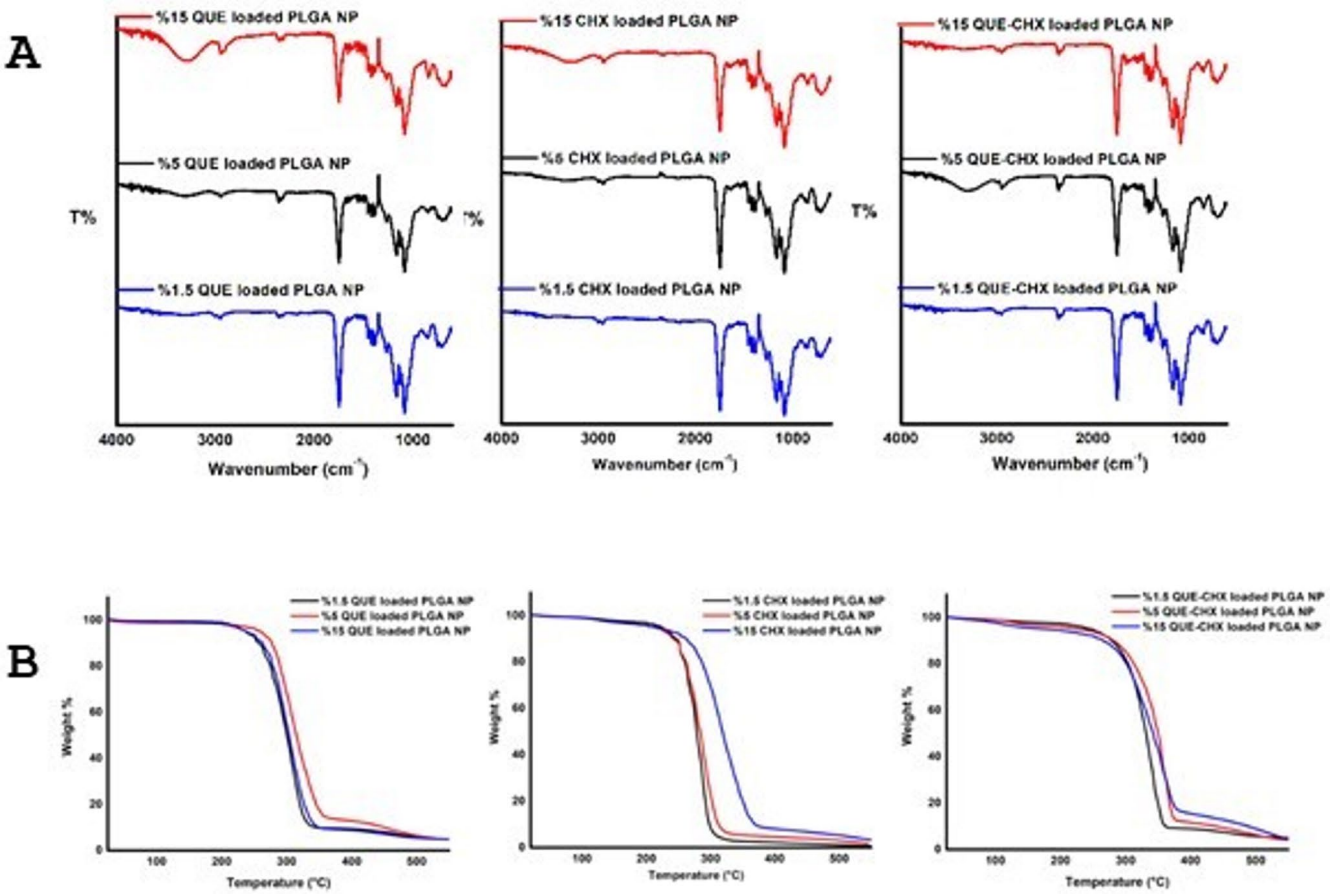
**(A)** FTIR spectra after 30 min UV irradiation of 1.5%, 5%, 15% QUE and/or CHX loaded PLGA NP, **(B)** TG thermograms after 30 min UV irradiation of 1.5%, 5%, 15% QUE and/or CHX loaded PLGA NP

CHX-loaded PLGANP, QUE-loaded PLGANP, and CHX/QUE-loaded PLGANP systems were sterilized under a UV lamp (254 nm) in a sterile cabinet for 30 min. FTIR and TG analyses were performed to examine degradation after the sterilization process. The obtained FTIR spectra and TG thermograms are shown in Fig. 3A and B, respectively. Following UV sterilization (254 nm, 30 min), the FTIR spectra and TG profiles of CHX-loaded PLGA NP, QUE-loaded PLGA NP, and CHX/QUE-loaded PLGA NP systems (Fig. 3A and B) showed comparable spectral features and thermal profiles.

### Antimicrobial activity of CHX and QUE-loaded PLGA nanoparticles

The effectiveness of the prepared nanoparticle systems against *E. coli* and *S. aureus* was investigated using antimicrobial susceptibility, minimum inhibitory concentration, and minimum bactericidal concentration assays, and the results are summarized in Table 2,^1^. In the agar diffusion assay, higher MEC values were observed for CHX/QUE-loaded PLGA nanoparticles and CHX-loaded PLGA nanoparticles compared to free CHX and QUE formulations. Based on these observations, subsequent antimicrobial evaluation was performed using MIC and MBC assays. CHX- and QUE-loaded formulations exhibited comparable MIC and MBC values against Gram-positive and Gram-negative bacteria.

**Table 2:** Antimicrobial efficacy test results

	*Escherichia coli*			*Staphylococcus aureus*		
Sample Code	MEC (𝜇g/mL)	MIC (𝜇g/mL)	MBC (𝜇g/mL)	MEC (𝜇g/mL)	MIC (𝜇g/mL)	MBC (𝜇g/mL)
CHX/QUE loaded PLGA NP	78	29	31	937	42	83
CHX loaded PLGA NP	156	25	83	781	42	75
QUE loaded PLGA NP	> 5000	> 1800	/	> 5000	> 1800	/
CHX	25	1,3	7	25	1,3	3
QUE	2500	> 1800	/	> 5000	> 1800	/

*MEC* Minimum concentration showing effect on agar plates; *MIC* Minimum inhibitory concentration; *MBC* Minimum bactericidal concentration; *CHX* Chlorhexidine; *QUE* Quercetin; *NP* Nanoparticles

## Discussion

The present study aimed to develop and characterize a dual-drug–loaded PLGA nanoparticle system incorporating quercetin (QUE) and chlorhexidine (CHX) for potential application in biofilm-associated pathologies. Comprehensive physicochemical characterization, in vitro release profiling, and antimicrobial evaluations were performed to assess the formulation’s performance, providing novel insights into the synergistic behavior of co-encapsulated bioactive agents within a polymeric delivery matrix.

FTIR analysis provided valuable insights into the molecular environment of the encapsulated compounds. In the FTIR spectra of QUE-loaded PLGA nanoparticles, the broadening and slight shifting of the O–H stretching bands with increasing QUE concentration indicate the possible formation of hydrogen-bonding interactions between quercetin and the PLGA matrix [51]. Similarly, the presence of characteristic N–H and C–N absorption bands in CHX-loaded PLGA nanoparticles is consistent with successful CHX incorporation, in agreement with previous reports.

For the dual-loaded PLGA nanoparticles, the concurrent detection of characteristic FTIR bands associated with both QUE (C = O stretching at ~ 1662 cm⁻¹) and CHX (C–N stretching at ~ 1093 cm⁻¹) confirms the successful co-encapsulation of both active agents within the same polymeric carrier system. Such molecular-level interactions may enhance the stability of the bioactive compounds within the polymer matrix and modulate their release behavior, as discussed in subsequent sections.The nanoparticle size was maintained below 200 nm, a range considered optimal for effective tissue penetration and retention at local application sites such as periodontal pockets. Nanoparticles within this size range are sufficiently small to diffuse through the extracellular matrix and interact efficiently with bacterial biofilms, while remaining large enough to avoid rapid clearance or systemic absorption [52].

The zeta potential values of the prepared nanoparticles ranged from − 2.11 to − 12.46 mV. Although relatively low in absolute magnitude, these values are regarded as adequate to ensure colloidal stability under physiological conditions, particularly in the presence of steric stabilizers such as PLGA and poly(vinyl alcohol) (PVA). As reported by Graf et al. (2012), even zeta potential values around ± 10 mV can effectively prevent particle aggregation and maintain dispersion in biological media when combined with appropriate surface functionalization and polymeric coating strategies. Furthermore, other studies have demonstrated that zeta potential values between − 10 and − 30 mV provide a favorable balance between colloidal stability and reduced cytotoxicity, making them suitable for biomedical applications such as drug delivery systems [52, 53]. In this context, the measured zeta potential values support the suitability of the developed nanoparticle system for localized delivery in biofilm-associated bacterial environments.

Thermogravimetric analysis (TGA) provided further insight into the thermal stability of the developed nanoparticle systems. Previous studies have shown that pure quercetin shows an initial mass loss at relatively low temperatures, followed by major thermal degradation above ~ 200 °C [54]. In contrast, PLGA-based nanostructures exhibit a single-step thermal degradation profile in the range of approximately 240–365 °C, with negligible mass loss at higher temperatures [55]. Moreover, CHX-loaded polymeric nanoparticle systems have been reported in the literature, indicating that incorporation of CHX into polymeric matrices can influence the thermal behavior and stability of the resulting formulations [56].

In the present study, TG thermograms of QUE-, CHX-, and CHX/QUE-loaded PLGA nanoparticles exhibited a single-step degradation profile with higher onset temperatures compared to free QUE. The observed enhancement in thermal stability and the relatively low residual mass values indicate that encapsulation within the PLGA matrix improves the thermal resistance of the active compounds. This stabilization effect may be attributed to restricted molecular mobility and polymer–drug interactions within the nanoparticle structure. DSC thermograms of the CHX/QUE-loaded PLGA nanoparticle systems revealed a broad endothermic peak spanning approximately 150 °C to 380 °C (Fig. 1C). Previous studies have reported that pure quercetin exhibits two distinct endothermic transitions, corresponding to the loss of bound water at around 130–140 °C and melting followed by decomposition at approximately 320–325 °C [57, 58]. In addition, free chlorhexidine has been shown to display an endothermic melting peak near 186 °C in DSC thermograms [59]. In contrast, the CHX/QUE-loaded PLGA nanoparticles in the present study demonstrated a single broad endothermic transition. This behavior is likely attributable to the shifting and overlapping of individual thermal events resulting from the incorporation of QUE and CHX into the PLGA matrix. Furthermore, the shift of the endothermic peak toward higher temperatures, approaching 350 °C, suggests the presence of intermolecular interactions both between QUE and CHX and between the drugs and the PLGA polymer.These interactions may contribute to the enhanced thermal stability of the encapsulated nanoparticle system.

Scanning electron microscopy (SEM) analysis demonstrated that the developed nanoparticles possessed a uniform and spherical morphology. Such structural characteristics are known to enhance colloidal stability and promote reproducible drug-release kinetics. Uniform spherical nanoparticles are commonly associated with predictable diffusion pathways and controlled erosion or degradation profiles, which together influence in vivo pharmacokinetic behavior. Previous studies have shown that the surface morphology of polymeric carriers plays a critical role in drug release, with smooth and spherical particles exhibiting more sustained and consistent release profiles due to reduced surface irregularities and improved particle packing behavior [60, 61]. Because nanoparticle shape and surface characteristics directly affect drug dissolution, absorption, and ultimately bioavailability, the homogeneous spherical morphology observed in our SEM micrographs is likely favorable for achieving controlled release and predictable pharmacokinetic performance [62]. Consistent with these observations, the presence of chlorine in the EDX spectra confirmed successful CHX incorporation, while carbon and oxygen elemental mapping supported the coexistence of both the polymeric matrix and the polyphenolic drug quercetin (QUE) within the nanoparticle system.

Drug release studies of CHX- and QUE-loaded nanoparticle systems revealed distinct release behaviors for the two active agents within the PLGA matrix. CHX exhibited a consistent and sustained release profile, reaching approximately 80% cumulative release in both single- and dual-drug formulations [63]. This behavior can be attributed to the relatively weak interactions between CHX and the PLGA matrix, together with its higher aqueous solubility, which facilitate diffusion through the polymeric network. In contrast, QUE displayed a more restricted release profile, likely due to its polyphenolic structure and stronger affinity for PLGA chains through hydrogen bonding and hydrophobic interactions [51, 64]. The formulation strategy markedly influenced QUE release kinetics. In single-loaded PLGA nanoparticles containing only QUE, drug release plateaued after 168 h, probably as a result of rapid depletion or degradation of the encapsulated compound. However, in dual-loaded systems incorporating CHX alongside QUE, detectable amounts of QUE persisted beyond 240 h, with approximately 20% of the drug remaining.This observation suggests a stabilizing or matrix-modulating effect of CHX, potentially through alterations in PLGA hydration, porosity, or degradation kinetics. Similar phenomena have been reported in co-encapsulation systems, where drug–drug and drug–polymer interactions lead to modified release kinetics and prolonged therapeutic duration [4, 51]. Furthermore, the physicochemical properties of CHX, including its limited aqueous solubility, may influence the microenvironment within the polymeric core and contribute to altered diffusion behavior. Collectively, these findings highlight the potential of dual-drug delivery platforms to achieve sustained and controlled release through matrix interactions and physicochemical modulation, thereby enhancing therapeutic performance in biofilm-associated pathologies [4, 51].

In addition to release behavior, antimicrobial activity was evaluated to assess the therapeutic potential of the developed formulations. CHX- and CHX/QUE-loaded nanoparticles demonstrated significantly greater antibacterial activity against both Gram-positive (*Staphylococcus aureus*) and Gram-negative (*Escherichia coli*) strains compared with QUE-only formulations. Although the CHX/QUE-loaded nanoparticles showed slightly lower immediate antibacterial activity than free CHX, their sustained release profiles are expected to provide prolonged local antimicrobial effects in oral biofilm-associated bacterial environments. Based on these observations, no clear dependence on bacterial cell wall type was evident under the experimental conditions. We acknowledge that periodontal pathogens such as *Porphyromonas gingivalis* are more directly implicated in periodontal disease. However, the primary objective of this study was to characterize the nanoparticle delivery system and to demonstrate the sustained antimicrobial activity of the encapsulated compounds. *E. coli* (Gram-negative) and *S. aureus* (Gram-positive) were therefore selected as representative model organisms due to their well-established growth characteristics and widespread use in antimicrobial screening studies.

Our findings further indicate the presence of drug–drug interactions at the polymer interface that influence matrix dynamics and release kinetics, a phenomenon previously reported in co-encapsulated nanocarrier systems. The incorporation of a more hydrophilic molecule such as CHX may promote polymer swelling or accelerate localized PLGA degradation, thereby enabling prolonged and controlled release of hydrophobic agents such as QUE. This observation is consistent with earlier reports demonstrating that the co-encapsulation of hydrophilic and hydrophobic drugs can modulate polymer porosity and reciprocally influence release kinetics within PLGA matrices [4].

Hydrophilic CHX salts may modify the local aqueous microenvironment and indirectly regulate diffusion and degradation pathways of co-encapsulated compounds [63]. Collectively, drug–drug and drug-polymer interactions in co-loaded nanoparticle systems can be exploited to design tunable release platforms capable of achieving synergistic therapeutic effects. These interactions are likely to affect matrix porosity, degradation rate, and drug–polymer affinity, thereby modulating both the release behavior and stability of the encapsulated agents [4, 63]. Such mechanisms have also been associated with enhanced therapeutic synergy and controlled co-release in polymer-based nanocarrier systems [51].

## Conclusion

These findings support the use of PLGA-based nanoparticles as effective carriers for dual-agent delivery, providing both antimicrobial and antioxidant functionalities. The co-encapsulation strategy not only optimizes release kinetics but also enhances the potential for sustained therapeutic activity. Collectively, these results justify further in vivo investigations to fully elucidate the clinical applicability of the CHX/QUE nanoparticle system in periodontal therapy and other localized infectious conditions.

## Data Availability

Data will be made available upon reasonable request.

## Footnotes

1 Table 2. Antimicrobial efficacy of free chlorhexidine (CHX), free quercetin (QUE), and their PLGA nanoparticle formulations against E. coli and S. aureus.
